# Association of direct bilirubin to total bilirubin ratio with 90-day mortality in patients with acute-on-chronic liver failure

**DOI:** 10.3389/fmed.2023.1286510

**Published:** 2023-11-09

**Authors:** Yuanji Ma, Lingyao Du, Shaoqun Zhou, Lang Bai, Hong Tang

**Affiliations:** Center of Infectious Diseases, West China Hospital of Sichuan University, Chengdu, China

**Keywords:** acute-on-chronic liver failure, direct bilirubin to total bilirubin ratio, prognosis, hepatic encephalopathy, risk factor

## Abstract

**Background:**

Hyperbilirubinemia occurs when the liver fails to process bilirubin properly. A disproportionate increase in direct bilirubin indicates a decreased ability of the hepatocytes to uptake and/or convert bilirubin, which may impact the prognosis of patients with acute-on-chronic liver failure (ACLF). However, the association of direct bilirubin to total bilirubin ratio (DB/TB) with outcomes in patients with ACLF remains unclear.

**Methods:**

A retrospective study was conducted in West China Hospital of Sichuan University to assess the association between DB/TB and 90-day mortality in patients with ACLF. The diagnosis of ACLF was based on the Chinese Group on the Study of Severe Hepatitis B (COSSH) ACLF criteria. Ordinal logistic regression models, linear regression models, and Cox proportional hazards models were applied to evaluate the association between DB/TB and hepatic encephalopathy, disease severity, and outcome, respectively.

**Results:**

A total of 258 patients with ACLF were included. The surviving patients were less likely to have liver cirrhosis and comorbidities, and their disease severities were milder than the dead. DB/TB was negatively correlated to cerebral score for hepatic encephalopathy (adjusted odds ratio: 0.01, *p* = 0.043), and disease severity (adjusted standardized coefficients: −0.42~−0.31, all *p* < 0.001), respectively. A significant 90-day mortality risk of DB/TB was observed [all adjusted hazard ratio (aHR) < 0.20 and all *p* ≤ 0.001]. Compared with patients with DB/TB < 0.80, patients with ACLF and DB/TB ≥ 0.80 had much lower 90-day mortality risk (all aHR < 0.75 and all *p* < 0.01).

**Conclusion:**

DB/TB could be an independent risk factor to predict the short-term prognosis in patients with ACLF. More attention should be paid to patients with lower DB/TB due to their poorer prognosis and more urgent need for liver transplantation.

**Clinical trial registration:**http://www.chictr.org.cn/showproj.aspx?proj=56960, identifier, ChiCTR2000035013.

## Introduction

Acute-on-chronic liver failure (ACLF) is a severe syndrome that is characterized by an abrupt worsening of clinical conditions in patients with chronic liver disease or liver cirrhosis (1). ACLF is life-threatening and associated with increased short-term mortality (2–5). Hyperbilirubinemia is not only one of the defining features of ACLF but also associated with disease severity and patient outcomes (2–4).

Under normal circumstances, circulating indirect bilirubin is taken up by hepatocytes and converted to direct bilirubin, which is then secreted into canalicular bile and emptied into the intestine (2). Dysfunction in any step of this process could result in hyperbilirubinemia; however, the alternation in the proportion of direct bilirubin suggests different pathophysiological mechanisms. Both total bilirubin and direct bilirubin are valuable for assessing disease severity and prognosis in patients with liver diseases (3). For Instance, hyperbilirubinemia is a well-documented neurotoxin in infants (4, 5), and direct bilirubin to total bilirubin ratio (DB/TB) >0.2 could be used as an aid in diagnosing cholestatic liver disease in infants aged 0 ~ 60 days (6). Previous case reports found that no proportional rise in direct bilirubin occurred in two patients with fulminant hepatic failure and the percentage of direct bilirubin declined progressively up to their deaths (7). In patients with acute liver injury caused by wild mushrooms, the indirect/direct bilirubin ratio, calculated differently but with similar implication, was significantly higher in non-survivors compared to survivors (8). These findings seem that, to a certain extent, the lower the proportion of direct bilirubin in total bilirubin in patients with liver failure, the higher the mortality risk. However, the relationship between DB/TB and the clinical outcomes of patients with ACLF has not been well described. Here, we conducted a retrospective study to assess the association between DB/TB and 90-day mortality of hepatitis B virus (HBV)-related ACLF (HBV-ACLF).

## Materials and methods

### Study design

A secondary data analysis was conducted based on a cohort of previously studied patients with ACLF and their medical records (9) at the Center of Infectious Diseases, West China Hospital of Sichuan University to assess the association between DB/TB and 90-day mortality of patients with HBV-ACLF. The cohort was registered with ChiCTR2000035013 after acquiring ethical approval from the Biomedical Research Ethics Committee of West China Hospital of Sichuan University (2020-650). All study components were performed according to the ethical standards laid down in the 1964 Declaration of Helsinki and its later amendments. Informed consent was obtained from all participants or, if the participants were under 18 years of age, from a parent and/or legal guardian.

### Patients

**Figure 1:**
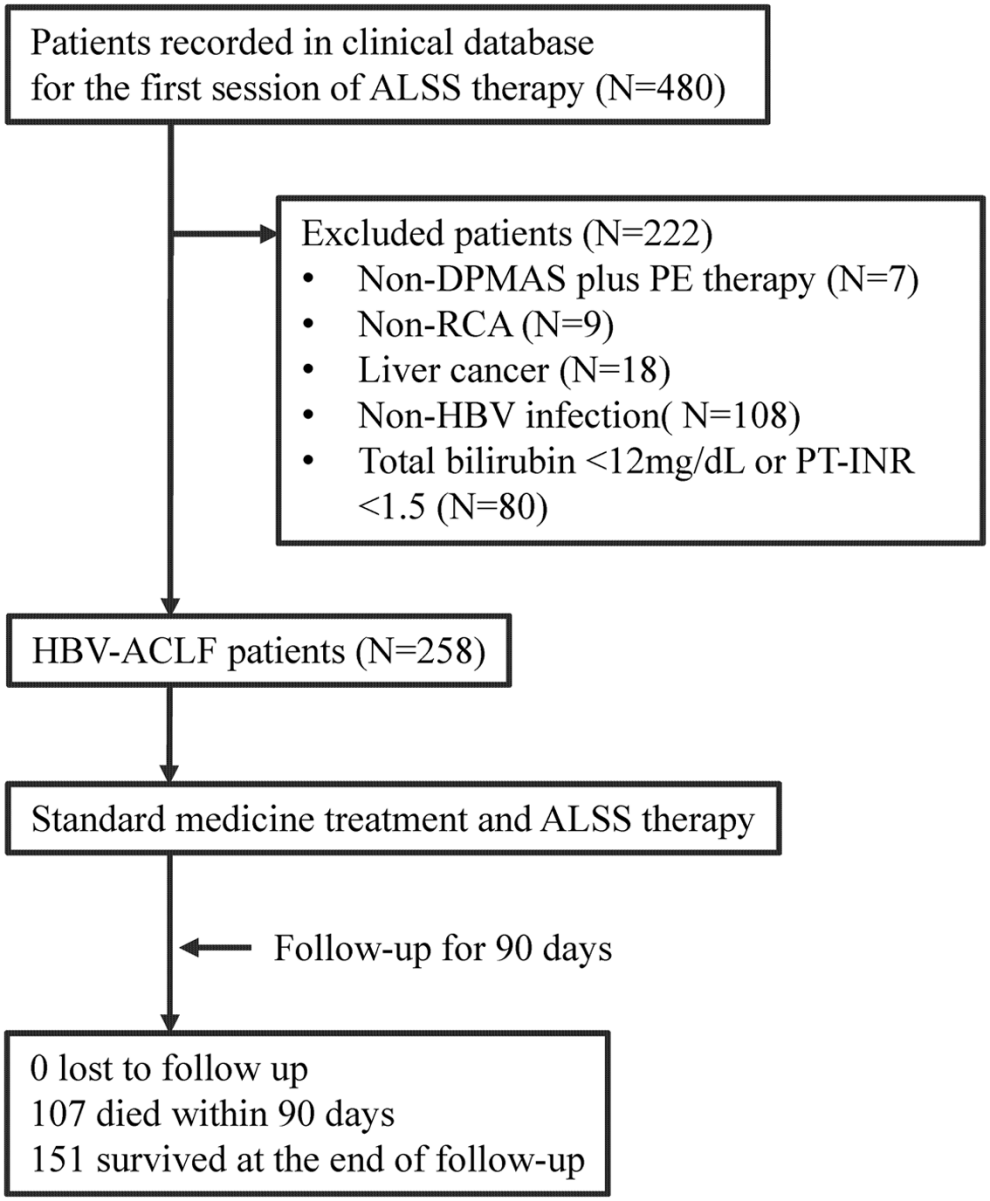
Flow diagram of patient selection and study process. ALSS, artificial liver support system; DPMAS, double plasma molecular adsorption system; PE, plasma exchange; RCA, regional citrate anticoagulation; HBV, hepatitis B virus; ACLF, acute-on-chronic liver failure; PT-INR, international normalized ratio (INR) of prothrombin time (PT).

The patients treated with artificial liver support system (ALSS) therapy were initially screened (Figure 1). The patients were excluded if they did not receive DPMAS plus PE therapy with regional citrate anticoagulation. The patients with liver cancer were also excluded. The remaining patients who fulfilled the diagnosis of HBV-ACLF were included.

HBV-ACLF was diagnosed according to the Chinese Group on the Study of Severe Hepatitis B (COSSH) ACLF criteria: regardless of the presence of cirrhosis, patients with chronic HBV infection, total bilirubin ≥ 12 mg/dL (205 μmol/L) and international normalized ratio of prothrombin time (PT-INR) ≥ 1.5 could be diagnosed with ACLF (10). The severity of HBV-ACLF was rated according to the COSSH ACLF score (10), COSSH ACLF II score (11), European Association for the Study of the Liver—Chronic Liver Failure-Consortium (CLIF-C) ACLF score (12), Asian Pacific Association for the Study of the Liver—ACLF Research Consortium (AARC) score (13), or Model for End-Stage Liver Disease (MELD) score (14). The diagnosis of liver cirrhosis was based on ultrasound and/or computed tomography (CT). Hepatic encephalopathy was defined as neuropsychiatric abnormalities, including the cognitive, affective, behavior and consciousness, and the brain edema was identified by CT (15). The cerebral score for hepatic encephalopathy was defined by the CLIF-C organ failure score system (12).

All patients received standard medical treatment and ALSS therapy (DPMAS plus PE therapy) with regional citrate anticoagulation. The standard medication included antiviral drugs, hepatoprotective agents, and drugs to treat complications and comorbidities. All patients received DPMAS therapy for 2 h, followed immediately by PE therapy with half the total plasma volume (approximately 1,500 mL) for approximately 1 h (16). The ALSS therapy was performed every 1 ~ 2 days and was discontinued due to one of the following conditions: improvement of patient’s condition and total bilirubin <10 mg/dL with reduced PT-INR, presence of conditions that did not allow further ALSS therapy, or patient refusal of receipt of further ALSS therapy (17).

### Statistical analysis

Quantitative data were represented as means ± standard deviation (SD; normally distributed data) or medians (interquartile ranges; non-normally distributed data) and compared by Mood’s median test. Qualitative data were represented as frequencies (proportion) and compared by the chi-squared test. The ordinal logistic regression model, linear regression models, and Cox proportional hazards regression models were applied to evaluate the association between DB/TB and hepatic encephalopathy, disease severity, and outcome, respectively. The optimal cut-off value of DB/TB was identified based on the area under the receiver operating characteristic curve (AUC) to separate the patients into groups with a low-risk and a high-risk of death. Propensity score matching analysis was used to generate compared pairs. The statistical tests mentioned above were performed using SPSS v.24 (IBM Corp.). Statistical significance was set at *p* < 0.05.

## Results

### Patient characteristics

From January 2018 to December 2019, a total of 258 patients who fulfilled the diagnostic criteria of HBV-ACLF were retrospectively enrolled and analyzed (Figure 1). Of these patients, the mean age was 46.2 ± 11.7 years, 37 (14.3%) patients were female, 202 (78.3%) patients had liver cirrhosis, and 107 (41.5%) patients died within the 90-day follow-up (Table 1). The surviving patients (*N* = 151) were less likely to have cirrhosis (70.9% vs. 88.8%, *p* = 0.001) and comorbidities (11.3% vs. 24.3%, *p* = 0.006), and their disease severity (COSSH ACLF score: 6.1 ± 0.7 vs. 7.1 ± 0.9, *p* < 0.001) was milder than the patients who died (*N* = 107). The DB/TB level among the surviving patients was much higher than that among those who died (0.80 ± 0.09 vs. 0.74 ± 0.09, *p* < 0.001).

**Table 1:** Characteristics of patients with HBV-ACLF.

	All patients (*N* = 258)	90-day prognosis		
		Mortality (*N* = 107)	Survival (*N* = 151)	*p*
Female	37(14.3%)	22(20.6%)	15(9.9%)	0.016
Age (years)	46.2 ± 11.7	49.2 ± 11.4	44.1 ± 11.5	0.058
Liver cirrhosis	202(78.3%)	95(88.8%)	107(70.9%)	0.001
HBV DNA (log10 IU/mL)	4.76(3.50~6.57)	4.68(3.47~6.26)	4.80(3.51~6.68)	0.800
Causes of liver disease				0.874
HBV infection only	194(75.2%)	81(75.7%)	113(74.8%)	
HBV infection plus other causes	64(24.8%)	26(24.3%)	38(25.2%)	
Comorbidities				0.006
No	215(83.3%)	81(75.7%)	134(88.7%)	
Yes	43(16.7%)	26(24.3%)	17(11.3%)	
**Disease severity assessment**				
COSSH ACLF score	6.5 ± 0.9	7.1 ± 0.9	6.1 ± 0.7	<0.001
COSSH ACLF II score	7.2 ± 0.8	7.7 ± 0.8	6.9 ± 0.7	<0.001
CLIF-C ACLF score	34.5 ± 7.2	38.1 ± 7.0	32.0 ± 6.2	<0.001
AARC score	9.9 ± 1.6	10.6 ± 1.4	9.4 ± 1.5	<0.001
MELD score	26.9 ± 4.8	29.3 ± 5.3	25.2 ± 3.6	<0.001
**Laboratory examination**				
PT-INR	2.12(1.77~2.53)	2.33(1.95~2.84)	2.02(1.73~2.33)	0.002
Serum creatinine (×ULN)	0.80(0.67~0.95)	0.88(0.72~1.13)	0.77(0.66~0.88)	0.001
Total bilirubin (μmol/L)	424.6 ± 124.6	479.4 ± 124.4	385.7 ± 109.5	<0.001
DB/TB	0.78 ± 0.10	0.74 ± 0.09	0.80 ± 0.09	<0.001
DB/TB ≥0.80	111(43.0%)	23(21.5%)	88(58.3%)	<0.001
Alanine aminotransferase (IU/L)	126(62~261)	118(58~232)	133(66~282)	0.613
Aspartate aminotransferase (IU/L)	118(83~198)	119(85~234)	117(79~191)	1.000
Aspartate aminotransferase to alanine aminotransferase ratio	1.08(0.64~1.67)	1.21(0.72~1.75)	0.97(0.59~1.49)	0.077
Albumin (g/L)	31.8 ± 3.9	31.2 ± 3.5	32.2 ± 4.0	0.055
Albumin to globulin ratio	1.2 ± 0.4	1.3 ± 0.5	1.2 ± 0.3	0.800
Ammonia (mmol/L)	79.1(60.0~111.3)	78.0(60.0~119.0)	80.2(60.0~108.0)	1.000
Lactate (mmol/L)	2.50(1.90~3.33)	2.80(2.20~3.87)	2.33(1.80~3.00)	0.006
Serum sodium (mmol/L)	133.4 ± 9.4	132.6 ± 5.1	133.9 ± 11.4	0.002
Serum potassium (mmol/L)	3.45 ± 0.60	3.48 ± 0.61	3.43 ± 0.54	0.848
Serum chloride (mmol/L)	96.3 ± 5.0	94.9 ± 5.9	97.4 ± 4.0	0.002
Hemoglobin (g/L)	118.8 ± 20.4	114.5 ± 21.2	121.9 ± 19.3	0.129
Platelets (×10^9^/L)	87(61~120)	77(48~113)	93(68~123)	0.070
White blood cells (×10^9^/L)	7.59 ± 3.66	8.59 ± 4.29	6.88 ± 2.96	0.002
ALSS therapy				
Sessions	4.0(3.0~6.0)	4.0(2.0~6.0)	4.0(3.0~6.0)	0.959
Days from the first to the last sessions	8.0(4.0~14.0)	8.0(4.0~14.0)	8.0(5.0~14.0)	0.566

HBV, hepatitis B virus; ACLF, acute-on-chronic liver failure; COSSH, Chinese Group on the Study of Severe Hepatitis B; CLIF-C, European Association for the Study of the Liver—Chronic Liver Failure-Consortium; AARC, APASL ACLF Research Consortium; APASL, Asian Pacific Association for the Study of the Liver; MELD, Model for End-Stage Liver Disease; PT-INR, international normalized ratio (INR) of prothrombin time (PT); ULN, upper limit of normal; DB/TB, direct bilirubin to total bilirubin ratio; ALSS, artificial liver support system. Quantitative data are represented as mean ± SD (normally distributed data) or median (interquartile range; non-normally distributed data) and compared by Mood’s median test. Qualitative data are represented as frequencies (proportion) and compared by chi-squared test.

### Association of DB/TB with hepatic encephalopathy and disease severity

DB/TB was negatively correlated to the cerebral score for hepatic encephalopathy [crude odds ratio (OR; 95% confidence interval (CI)), 0.00 (0.00~0.06), *p* = 0.001]. A significant correlation of DB/TB with cerebral score was also observed with an ordinal logistic regression model that was established with DB/TB, age, sex, liver cirrhosis, HBV DNA, other co-existing liver diseases, comorbidities, disease severity (MELD score), and venous ammonia [adjusted OR (aOR; 95% CI), 0.01 (0.00~0.86), *p* = 0.043].

**Figure 2:**
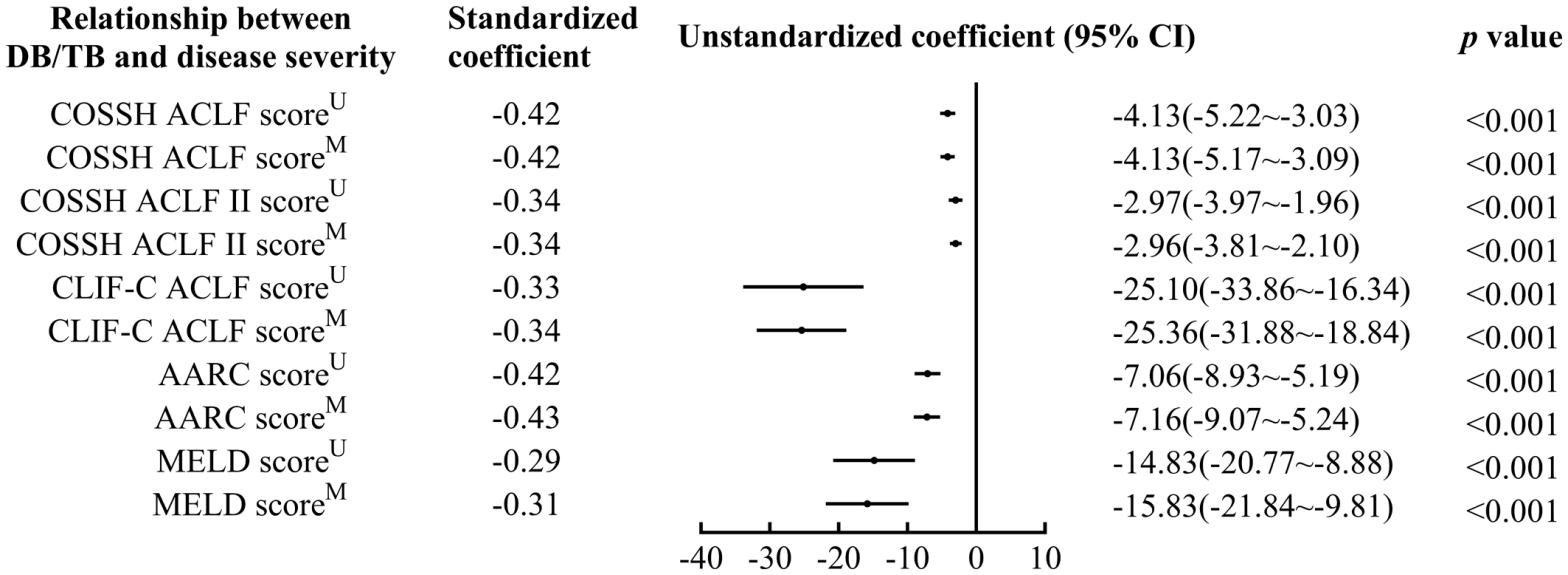
Relationship between DB/TB and disease severity in patients with ACLF. DB/TB, direct bilirubin to total bilirubin ratio; ACLF, acute-on-chronic liver failure; HBV, hepatitis B virus; COSSH, Chinese Group on the Study of Severe Hepatitis B; CLIF-C, European Association for the Study of the Liver—Chronic Liver Failure-Consortium; AARC, APASL ACLF Research Consortium; APASL, Asian Pacific Association for the Study of the Liver; MELD, Model for End-Stage Liver Disease; CI, confidence interval. ^U^, Univariate linear regression analysis. ^M^, Multivariate linear regression analysis includes DB/TB (continuous values), age (continuous years), gender (female vs. male), liver cirrhosis (yes vs. no), HBV DNA (continuous log10 IU/mL), other co-existing liver diseases (yes vs. no), comorbidities (yes vs. no).

DB/TB was negatively correlated with disease severity (Standardized coefficients for COSSH ACLF score, COSSH ACLF II score, CLIF-C ACLF score, AARC score, and MELD score: −0.42~−0.29, all *p* < 0.001; Figure 2; Supplementary Table 1). A significant correlation of DB/TB with disease severity was also observed with the linear regression models that were established with DB/TB, age, sex, liver cirrhosis, HBV DNA, other co-existing liver diseases, comorbidities, and disease severity (Model 1, COSSH ACLF score; Model 2, COSSH ACLF II score; Model 3, CLIF-C ACLF score; Model 4, AARC score; Model 5, MELD score; adjusted standardized coefficients for Model 1~5, −0.42~−0.31, all *p* < 0.001).

### Association of DB/TB with outcome

**Figure 3:**
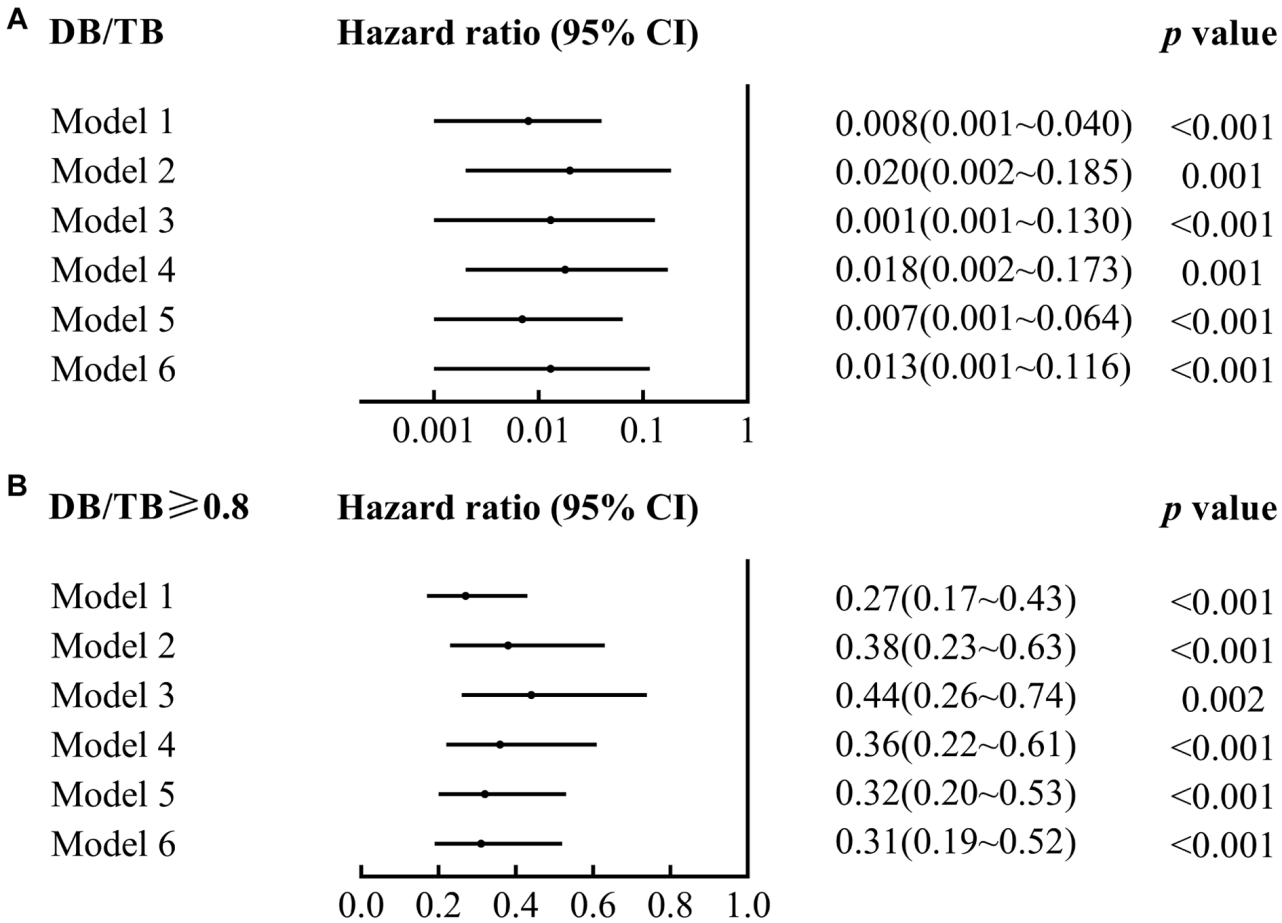
Association of DB/TB and DB/TB ≥0.80 with 90-day mortality in patients with ACLF. DB/TB, direct bilirubin to total bilirubin ratio; CI, confidence interval. Model 1, Univariate Cox regression analysis. Model 2~6, Multivariate Cox regression analysis includes DB/TB (continuous values; **A**) or DB/TB ≥0.80 (yes vs. no; **B**), age (continuous years), gender (female vs. male), liver cirrhosis (yes vs. no), HBV DNA (continuous log10 IU/mL), other co-existing liver diseases (yes vs. no), comorbidities (yes vs. no), disease severity (Model 2, COSSH ACLF score; Model 3, COSSH ACLF II score; Model 4, CLIF-C ACLF score; Model 5, AARC score; Model 6, MELD score), and ALSS therapy sessions (continuous values).

DB/TB was a risk factor for 90-day mortality in patients with ACLF (crude hazard ratio (HR; 95% CI), 0.01 (0.00~0.04), *p* < 0.001; Model 1 in Figure 3A; Supplementary Table 2). A significant 90-day mortality risk of DB/TB was also observed with the Cox proportional hazards models that were established with DB/TB, age, sex, liver cirrhosis, HBV DNA, other co-existing liver diseases, comorbidities, disease severity (Model 2, COSSH ACLF score; Model 3, COSSH ACLF II score; Model 4, CLIF-C ACLF score; Model 5, AARC score; Model 6, MELD score), and sessions of ALSS therapy [adjusted HR (aHR; 95% CI) for Model 2, 0.02 (0.00~0.19), *p* = 0.001; Model 3, 0.01 (0.00~0.13), *p* < 0.001; Model 4, 0.02 (0.00~0.17), *p* = 0.001; Model 5, 0.01 (0.00~0.06), *p* < 0.001; Model 6, 0.01 (0.00~0.05), *p* < 0.001; Model 2 ~ 6 in Figure 3A; Supplementary Table 2].

**Figure 4:**
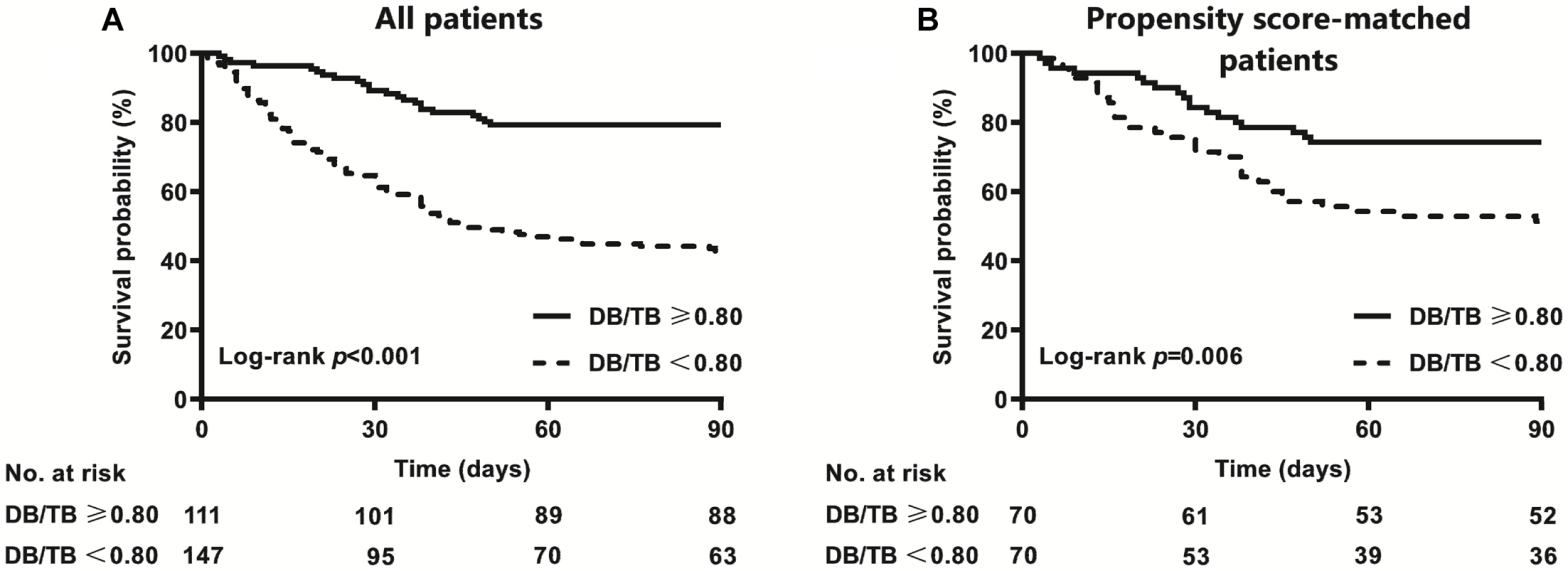
Survival curves of patients with ACLF with or without DB/TB ≥ 0.80 [**(A)** all patients; **(B)** propensity score-matched patients] direct bilirubin to total bilirubin ratio. Propensity score matching analysis includes age (continuous years), gender (female vs. male), liver cirrhosis (yes vs. no), HBV DNA (continuous log10 IU/mL), other co-existing liver diseases (yes vs. no), comorbidities (yes vs. no), disease severity (COSSH ACLF score), and ALSS therapy sessions (continuous values).

The AUC of DB/TB in predicting 90-day survival was 0.706 (0.642~0.769; *p* < 0.001). The best cut-off value for DB/TB was 0.80, and the sensitivity and specificity were 58.3% and 80.4%, respectively. The 90-day mortality of patients with ACLF and DB/TB ≥ 0.80 (*N* = 111) was much lower than that of patients with DB/TB < 0.80 (*N* = 147; 20.7% vs. 57.1%, log-rank *p* < 0.001; Figure 4A). Lower 90-day mortality of patients with ACLF and DB/TB ≥ 0.80 (*N* = 70) than that of patients with DB/TB < 0.80 (*N* = 70) was also observed based on propensity score matching analysis using age, sex, liver cirrhosis, HBV DNA, other co-existing liver diseases, comorbidities, disease severity (COSSH ACLF score) and sessions of ALSS therapy (25.7% vs. 48.6%, log-rank *p* = 0.006; Figure 4B).

Compared with patients with ACLF and DB/TB < 0.80, patients with DB/TB ≥ 0.80 had much lower 90-day mortality risk [crude HR (95% CI), 0.27 (0.17~0.43), *p* < 0.001; Model 1 in Figure 3B; Supplementary Table 3]. Similarly, significant 90-day mortality risk of DB/TB ≥ 0.80 was also observed with similar Cox models that were established with DB/TB ≥ 0.80, age, sex, liver cirrhosis, HBV DNA, other co-existing liver diseases, comorbidities, disease severity (Model 2, COSSH ACLF score; Model 3, COSSH ACLF II score; Model 4, CLIF-C ACLF score; Model 5, AARC score; Model 6, MELD score), and sessions of ALSS therapy [aHR (95% CI) for Model 2, 0.38 (0.23~0.63), *p* < 0.001; Model 3, 0.44 (0.26~0.74), *p* = 0.002; Model 4, 0.36 (0.22~0.61), *p* < 0.001; Model 5, 1.060.32 (0.20~0.53), *p* < 0.001; Model 6, 0.31 (0.19~0.52), *p* < 0.001; Model 2~6 in Figure 3B; Supplementary Table 3].

## Discussion

Bilirubin is an endogenous end product of blood catabolism, and mildly elevated bilirubin is considered a protective bioactive molecule with anti-oxidation, anti-inflammatory, and other vital physiological functions (18). However, hyperbilirubinemia is a well-documented neurotoxin in infants (4, 5), and is one of the defining features of ACLF (10, 11, 13). Both total bilirubin and direct bilirubin are valuable for assessing disease severity and prognosis in patients with liver diseases, and direct bilirubin is more valuable than total bilirubin in patients with liver cirrhosis (3). In this retrospective study, we found that DB/TB is negatively related to hepatic encephalopathy and disease severity in patients with ACLF. The DB/TB is an independent risk factor to predict short-term prognosis.

Hepatocytes are primarily responsible for metabolism and excretion of bilirubin. The disproportionate increase in direct bilirubin implies hepatocytes dysfunction in the early stages, failing to take up and/or convert indirect bilirubin and to a deeper degree. Therefore, the DB/TB could reflect the ability of bilirubin processing during liver failure to a certain extent. However, few previous reports have observed the correlation between DB/TB and the outcomes of patients with liver failure. In this study, we found that DB/TB is an independent risk factor to predict short-term prognosis (all aHR < 0.20 and all *p* < 0.001), and patients with ACLF and lower DB/TB experienced an increased risk of 90-day mortality (all aHR < 1 and all *p* < 0.01). The direct bilirubin to indirect bilirubin ratio (DIR), which is applied to predict prognosis in patients with acute coronary syndrome or colorectal cancer (19, 20), is calculated differently but has similar implications and might have similar values. In a study to identify early predictive markers of poor outcomes in patients with wild mushroom intoxication, the indirect/direct bilirubin ratio, the inverse of DIR, was significantly higher in non-survivors compared to survivors (2.45 ± 1.39 vs. 0.99 ± 0.45, *p* < 0.01) (8). The study population was acute liver injury, defined by a > 5-fold elevation of liver enzymes or moderate coagulopathy (PT-INR > 2.0), while ours was patients with HBV-ACLF; although the characteristics of the participants were different, the main finding is consistent. In clinical practice, more attention should be paid to patients with ACLF and low DB/TB, low DIR, or high indirect/direct bilirubin ratio, especially to those with progressive decline of DB/TB and a gradual increase in indirect bilirubin, because of their poorer prognosis and more urgent need for liver transplantation.

DB/TB and DIR have also been used in the diagnosis of other diseases or evaluation of patient prognosis. DB/TB > 0.5 was associated with poor clinical outcomes, such as mortality, in hospitalized patients who were diagnosed with COVID-19 pneumonia (21). The DIR was positively correlated with poor prognosis in acute coronary syndrome (19), and was associated with poor clinical outcomes in patients with colorectal cancer with a best cutoff value of 0.42 (20).

Indirect bilirubin is combined with albumin and then transported in the blood. The part of indirect bilirubin that is not bounded by albumin is referred to as free bilirubin. The free bilirubin is fat-soluble and can penetrate cell membranes, but with extremely low content, it is harmless to healthy humans. In liver failure, the content of free bilirubin increases due to increased indirect bilirubin, insufficient quantity and/or, decreased binding capacity of albumin (22). Previous clinical and basic studies have found that free bilirubin in serum is an important cause of kernicterus in neonates and adults (4, 5, 23, 24). Recently, a retrospective case–control study showed that the indirect bilirubin-albumin ratio is an independent factor of hepatic encephalopathy in patients with liver failure (aOR:1.63, 95% CI: 1.32~2.00, *p* < 0.001) (25). Our finding that DB/TB is negatively correlated to cerebral score for hepatic encephalopathy (aOR: 0.01, 95% CI: 0.00~0.86, *p* = 0.043) is in accordance with these results. Notably, the administration of albumin or by pharmacological inhibition of indirect bilirubin production in a genetic model of hyperbilirubinemia could improve neurodevelopment and reduce apoptosis of cerebral cells remarkably (25, 26). Similarly, promoting the conversion of indirect bilirubin to direct bilirubin was shown as beneficial in two patients with Crigler-Najjar syndrome type II who developed kernicterus in adulthood (24). Taken together, free bilirubin, DB/TB and indirect bilirubin-albumin ratio are risk factors for neurological dysfunction and nerve damage in patients with liver failure. Reducing free bilirubin, especially in those patients with low DB/TB, low DIR or a high indirect bilirubin-albumin ratio, may be a new strategy for the treatment of hepatic encephalopathy in patients with ACLF.

The short-term prognosis of patients with HBV-ACLF is extremely poor. Many therapeutic interventions have been developed to improve their prognosis. These therapeutic strategies, drugs or operations are consistently under study and optimization. ALSS therapy has been developed as an available method for patients with ACLF and is a bridge to liver transplantation. Several studies have shown that ALSS therapy could significantly improve the short-term prognosis of patients with ACLF (27), especially the PE-centered methods (28, 29). In this study, we found that the sessions of ALSS therapy were another independent risk factor for 90-day mortality in patients with HBV-ACLF (all aHR <1 and all *p* < 0.01). This finding is consistent with previous studies (17).

This study had limitations. First, as a monocentric retrospective study with a small number of patients, the patients’ characteristics may not represent the general population, which may have had a causal effect on the relationship of DB/TB with outcome. Second, the patients in our study were all HBV-ACLF cases and fulfilled the COSSH ACLF criteria (10). The results that were derived from this subset may not be applicable to patients who are diagnosed according to other ACLF criteria or patients without chronic HBV infection. Third, it is thought that PE-centered ALSS therapy could improve the short-term prognosis of patients with liver failure (28–30). All patients received PE-centered ALSS therapy in our study, which may also have affected the results.

In conclusion, our study could provide evidence that patients with ACLF and lower DB/TB experience an increased risk of 90-day mortality. More attention should be paid to patients with lower DB/TB due to their poorer prognoses and more urgent need for liver transplantation. Further large-scale, multi-center, prospective, cohort studies are warranted to evaluate the performance of DB/TB combined with other independent risk factors. A validated model with DB/TB would help to assess disease severity and predict outcomes in patients with ACLF and guide clinical management.

## Data availability statement

The original contributions presented in the study are included in the article/Supplementary material, further inquiries can be directed to the corresponding authors.

## Ethics statement

The studies involving humans were approved by the Biomedical Research Ethics Committee of West China Hospital of Sichuan University. The studies were conducted in accordance with the local legislation and institutional requirements. The participants provided their written informed consent to participate in this study.

## Author contributions

YM: Data curation, Formal analysis, Funding acquisition, Investigation, Methodology, Resources, Writing – original draft, Writing – review & editing. LD: Data curation, Formal analysis, Funding acquisition, Investigation, Writing – original draft, Writing – review & editing. SZ: Conceptualization, Funding acquisition, Methodology, Project administration, Writing – review & editing. LB: Conceptualization, Funding acquisition, Methodology, Project administration, Resources, Supervision, Writing – review & editing, HT: Conceptualization, Funding acquisition, Project administration, Supervision, Writing – review & editing.
