# Supplementary material for: Association of direct bilirubin to total bilirubin ratio with 90-day mortality in patients with acute-on-chronic liver failure
Source: Front Med (Lausanne). 2023 Nov 9;10:1286510. doi: 10.3389/fmed.2023.1286510 (PMC10666058; doi:10.3389/fmed.2023.1286510)
Supplement: Supplementary file 1 [file Table_1.docx]

**Association of direct bilirubin to total bilirubin ratio with 90-day mortality in patients with acute-on-chronic liver failure**

Yuanji Ma, Lingyao Du, Shaoqun Zhou*, Lang Bai*, Hong Tang

Center of Infectious Diseases, West China Hospital of Sichuan University, Chengdu, China

***Correspondence:**

Lang Bai, pangbailang@163.com

Shaoqun Zhou, 253044247@qq.com

**Supplementary Table 1. Relationship between DB/TB and disease severity.**

| Disease severity | Univariate linear regression | |  | Multivariate linear regression^▲^ | |
| --- | --- | --- | --- | --- | --- |
|  | Unstandardized Coefficient (95% CI) | Standardized coefficient |  | Unstandardized Coefficient (95% CI) | Standardized coefficient |
| COSSH ACLF score | -4.13(-5.22~-3.03)^***^ | -0.42 |  | -4.13(-5.17~-3.09)^***^ | -0.42 |
| COSSH ACLF II score | -2.97(-3.97~-1.96)^***^ | -0.34 |  | -2.96(-3.81~-2.10)^***^ | -0.34 |
| CLIF-C ACLF score | -25.10(-33.86~-16.34)^***^ | -0.33 |  | -25.36(-31.88~-18.84)^***^ | -0.34 |
| AARC score | -7.06(-8.93~-5.19)^***^ | -0.42 |  | -7.16(-9.07~-5.24)^***^ | -0.43 |
| MELD score | -14.83(-20.77~-8.88)^***^ | -0.29 |  | -15.83(-21.84~-9.81)^***^ | -0.31 |

Abbreviations: DB/TB, direct bilirubin to total bilirubin ratio; ACLF, acute-on-chronic liver failure; HBV, hepatitis B virus; COSSH, Chinese Group on the Study of Severe Hepatitis B; CLIF-C, European Association for the Study of the Liver—Chronic Liver Failure-Consortium; AARC, APASL ACLF Research Consortium; APASL, Asian Pacific Association for the Study of the Liver; MELD, Model for End-Stage Liver Disease; CI, confidence interval.

^▲^: Multivariate linear regression analysis includes DB/TB (continuous values), age (continuous years), gender (female vs. male), liver cirrhosis (yes vs. no), HBV DNA (continuous log10 IU/mL), other co-existing liver diseases (yes vs. no), comorbidities (yes vs. no).

^***^*p*<0.001, ^**^*p*<0.01, ^*^*p*<0.05, the others *p*>0.05.

**Supplementary Table 2. DB/TB and other factors associated with 90-day mortality risk in ACLF patients.**

|  | Crude HR (95% CI) | Adjusted HR^▲^(95% CI) | | | | |
| --- | --- | --- | --- | --- | --- | --- |
|  |  | Model 1 | Model 2 | Model 3 | Model 4 | Model 5 |
| DB/TB | 0.008 (0.001~0.040)^***^ | 0.020 (0.002~0.185)^**^ | 0.013 (0.001~0.130)^***^ | 0.018 (0.002~0.173)^**^ | 0.007 (0.001~0.064)^***^ | 0.013 (0.001~0.116)^***^ |
| Age (years) | 1.03 (1.01~1.05)^***^ | 1.00 (0.98~1.02) | 0.97 (0.95~0.99)^*^ | 0.98 (0.95~1.00) | 1.02 (1.00~1.04)^*^ | 1.02 (1.00~1.04) |
| Gender |  |  |  |  |  |  |
| Male | 1 | 1 | 1 | 1 | 1 | 1 |
| Female | 1.84 (1.15~2.94)^*^ | 1.16 (0.70~1.94) | 1.49 (0.90~2.47) | 1.10 (0.66~1.82) | 1.36 (0.82~2.26) | 1.54 (0.92~2.56) |
| Liver cirrhosis |  |  |  |  |  |  |
| No | 1 | 1 | 1 | 1 | 1 | 1 |
| Yes | 2.51 (1.37~4.57)^**^ | 1.66 (0.90~3.07) | 1.95 (1.06~3.59)^*^ | 2.06 (1.12~3.80)^*^ | 2.14 (1.16~3.97)^*^ | 1.97 (1.07~3.63)^*^ |
| HBV DNA (log10 IU/mL) | 0.98 (0.89~1.09) | 1.04 (0.94~1.16) | 1.07 (0.96~1.20) | 1.03 (0.93~1.15) | 1.05 (0.94~1.18) | 1.04 (0.93~1.16) |
| Etiology |  |  |  |  |  |  |
| HBV infection only | 1 | 1 | 1 | 1 | 1 | 1 |
| HBV infection plus other causes^■^ | 0.93 (0.60~1.45) | 1.04 (0.66~1.63) | 1.01 (0.64~1.60) | 0.99 (0.62~1.57) | 1.10 (0.70~1.74) | 0.78 (0.49~1.24) |
| Comorbidity^◆^ |  |  |  |  |  |  |
| No | 1 | 1 | 1 | 1 | 1 | 1 |
| Yes | 1.86 (1.20~2.90)^**^ | 1.83 (1.11~3.04)^*^ | 2.03 (1.22~3.39)^*^ | 1.62 (0.99~2.65) | 1.71 (1.04~2.80)^*^ | 1.86 (1.12~3.10)^*^ |
| Disease severity |  |  |  |  |  |  |
| COSSH ACLF score | 2.78 (2.31~3.34)^***^ | 2.37 (1.92~2.93)^***^ | - | - | - | - |
| COSSH ACLF II score | 3.71 (2.85~4.82)^***^ | - | 3.67 (2.71~4.96)^***^ | - | - | - |
| CLIF-C ACLF score | 1.11 (1.08~1.14)^***^ | - | - | 1.13 (1.08~1.17)^***^ | - | - |
| AARC score | 1.60 (1.41~1.82)^***^ | - | - | - | 1.58 (1.38~1.81)^***^ | - |
| MELD score | 1.16 (1.12~1.20)^***^ | - | - | - | - | 1.14 (1.10~1.19)^***^ |
| ALSS therapy sessions | 0.93 (0.85~1.01) | 0.87 (0.80~0.95)^**^ | 0.85 (0.78~0.93)^**^ | 0.87 (0.79~0.95)^**^ | 0.82 (0.75~0.90)^***^ | 0.85 (0.78~0.93)^**^ |

Abbreviations: DB/TB, direct bilirubin to total bilirubin ratio; ACLF, acute-on-chronic liver failure; HBV, hepatitis B virus; COSSH, Chinese Group on the Study of Severe Hepatitis B; CLIF-C, European Association for the Study of the Liver—Chronic Liver Failure-Consortium; AARC, APASL ACLF Research Consortium; APASL, Asian Pacific Association for the Study of the Liver; MELD, Model for End-Stage Liver Disease; ALSS, artificial liver support system; HR, hazard ratio; CI, confidence interval.

HBV infection plus other causes^■^: the ones having HBV infection plus any one of other co-existing liver diseases was classified to this subgroup.

Comorbidity^◆^: the ones having any one of comorbidities was classified as the comorbidity group.

Adjusted HR^▲^: multivariable Cox regression analysis includes DB/TB (continuous values), age (continuous years), gender (female vs. male), liver cirrhosis (yes vs. no), HBV DNA (continuous log10 IU/mL), other co-existing liver diseases (yes vs. no), comorbidities (yes vs. no), disease severity (Model 1, COSSH ACLF score; Model 2, COSSH ACLF II score; Model 3, CLIF-C ACLF score; Model 4, AARC score; Model 5, MELD score), and ALSS therapy sessions (continuous values).

^***^*p*<0.001, ^**^*p*<0.01, ^*^*p*<0.05, the others *p*>0.05.

**Supplementary Table 3. DB/TB ≥0.80 and other factors associated with 90-day mortality risk in ACLF patients.**

|  | Crude HR (95% CI) | Adjusted HR^▲^(95% CI) | | | | |
| --- | --- | --- | --- | --- | --- | --- |
|  |  | Model 1 | Model 2 | Model 3 | Model 4 | Model 5 |
| DB/TB **≥**0.80 |  |  |  |  |  |  |
| No | 1 | 1 | 1 | 1 | 1 | 1 |
| Yes | 0.27 (0.17~0.43)^***^ | 0.38 (0.23~0.63)^***^ | 0.44 (0.26~0.74)^**^ | 0.36 (0.22~0.61)^***^ | 0.32 (0.20~0.53))^***^ | 0.31 (0.19~0.52)^***^ |
| Age (years) | 1.03 (1.01~1.05)^***^ | 1.00 (0.97~1.02) | 0.97 (0.95~0.99)^**^ | 0.97 (0.95~1.00)^*^ | 1.02 (1.00~1.04) | 1.01 (0.99~1.03) |
| Gender |  |  |  |  |  |  |
| Male | 1 | 1 | 1 | 1 | 1 | 1 |
| Female | 1.84 (1.15~2.94)^*^ | 1.32 (0.79~2.18) | 1.67 (1.01~2.75)^*^ | 1.30 (0.79~2.13) | 1.56 (0.94~2.57) | 1.73 (1.05~2.87)^*^ |
| Liver cirrhosis |  |  |  |  |  |  |
| No | 1 | 1 | 1 | 1 | 1 | 1 |
| Yes | 2.51 (1.37~4.57)^**^ | 1.79 (0.96~3.32) | 1.99 (1.08~3.68)^*^ | 2.18 (1.18~4.03)^*^ | 2.29 (1.22~4.28)^**^ | 2.10 (1.13~3.89)^*^ |
| HBV DNA (log10 IU/mL) | 0.98 (0.89~1.09) | 1.05 (0.94~1.17) | 1.07 (0.96~1.19) | 1.03 (0.92~1.14) | 1.04 (0.93~1.16) | 1.05 (0.93~1.17) |
| Etiology |  |  |  |  |  |  |
| HBV infection only | 1 | 1 | 1 | 1 | 1 | 1 |
| HBV infection plus other causes^■^ | 0.93 (0.60~1.45) | 1.32 (0.83~2.09) | 1.22 (0.77~1.93) | 1.24 (0.78~1.95) | 1.43 (0.90~2.29) | 1.05 (0.66~1.67) |
| Comorbidity^◆^ |  |  |  |  |  |  |
| No | 1 | 1 | 1 | 1 | 1 | 1 |
| Yes | 1.86 (1.20~2.90)^**^ | 1.83 (1.10~3.03)^*^ | 2.03 (1.22~3.37)^**^ | 1.61 (0.98~2.64) | 1.68 (1.02~2.76)^*^ | 1.87 (1.12~3.12)^*^ |
| Disease severity |  |  |  |  |  |  |
| COSSH ACLF score | 2.78 (2.31~3.34)^***^ | 2.34 (1.89~2.89)^***^ | - | - | - | - |
| COSSH ACLF II score | 3.71 (2.85~4.82)^***^ | - | 3.53 (2.57~4.84)^***^ | - | - | - |
| CLIF-C ACLF score | 1.11 (1.08~1.14)^***^ | - | - | 1.12 (1.07~1.17)^***^ | - | - |
| AARC score | 1.60 (1.41~1.82)^***^ | - | - | - | 1.55 (1.35~1.78)^***^ | - |
| MELD score | 1.16 (1.12~1.20)^***^ | - | - | - | - | 1.14 (1.10~1.18)^***^ |
| ALSS therapy sessions | 0.93 (0.85~1.01) | 0.88 (0.80~0.96)^**^ | 0.87 (0.80~0.95)^**^ | 0.87 (0.80~0.95)^**^ | 0.83 (0.76~0.91)^***^ | 0.86 (0.79~0.94)^**^ |

Abbreviations: DB/TB, direct bilirubin to total bilirubin ratio; ACLF, acute-on-chronic liver failure; HBV, hepatitis B virus; COSSH, Chinese Group on the Study of Severe Hepatitis B; CLIF-C, European Association for the Study of the Liver—Chronic Liver Failure-Consortium; AARC, APASL ACLF Research Consortium; APASL, Asian Pacific Association for the Study of the Liver; MELD, Model for End-Stage Liver Disease; ALSS, artificial liver support system; HR, hazard ratio; CI, confidence interval.

HBV infection plus other causes^■^: the ones having HBV infection plus any one of other co-existing liver diseases was classified to this subgroup.

Comorbidity^◆^: the ones having any one of comorbidities was classified as the comorbidity group.

Adjusted HR^▲^: multivariable Cox regression analysis includes DB/TB (≥0.80 vs. **<**0.80), age (continuous years), gender (female vs. male), liver cirrhosis (yes vs. no), HBV DNA (continuous log10 IU/mL), other co-existing liver diseases (yes vs. no), comorbidities (yes vs. no), disease severity (Model 1, COSSH ACLF score; Model 2, COSSH ACLF II score; Model 3, CLIF-C ACLF score; Model 4, AARC score; Model 5, MELD score), and ALSS therapy sessions (continuous values).

^***^*p*<0.001, ^**^*p*<0.01, ^*^*p*<0.05, the others *p*>0.05.
